# Context reexposure to bolster contextual dependency of emotional episodic memory

**DOI:** 10.1038/s41598-023-40982-0

**Published:** 2023-10-18

**Authors:** Wouter R. Cox, Mandy Woelk, Olivier T. de Vries, Angelos-Miltiadis Krypotos, Merel Kindt, Iris M. Engelhard, Dieuwke Sevenster, Vanessa A. van Ast

**Affiliations:** 1 Department of Clinical Psychology, University of Amsterdam, Amsterdam, The Netherlands; 2 Research Unit Behaviour, Health, and Psychopathology, KU Leuven, Leuven, Belgium; 3 Department of Clinical Psychology, Utrecht University, Utrecht, The Netherlands

**Keywords:** Psychology, Learning and memory

## Abstract

Contextual overgeneralization of emotional memory is a core aspect of anxiety disorders. Identifying methods to enhance contextual dependency of emotional memory is therefore of significant clinical interest. Animal research points to a promising approach: reexposure to the context in which fear is acquired reduces generalization to other contexts. However, the exact conditions for this effect are unknown, complicating translation to effective interventions. Most notably, exposure to a context that resembles—but is not identical to—the learning context may diminish contextual dependency of memory by integration of additional contextual cues. Here, we therefore assessed in a large-scale study (*N* = 180) whether context reexposure enhances contextual dependency of emotional episodic memory whereas exposure to a similar context impairs it. We also tested whether relatively strong memory retrieval during context (re)exposure amplifies these effects. We replicated prior research showing that correct recognition depends on context and contextual dependency is lower for emotional than neutral memories. However, exposure to the encoding context or a similar context did not affect contextual dependency of memory, and retrieval strength did not interact with such effects. Thorough insight into factors underlying the effects of context (re)exposure on contextual dependency seems key to eventually attain a memory recontextualization intervention.

## Introduction

When one returns to the spatial environment in which an event has previously occurred, retrieval or recognition of that event often happens readily, whereas memories can be more difficult to reinstate in different contexts^1–3^. Such contextual dependency of memory is, however, less pronounced for emotional compared to neutral memories, an observation that is partly attributed to relatively weak embedding of emotional events in their encoding context^4–6^. The reduced contextual dependency of emotional memory is believed to lie at the heart of dysfunctional memory development, resulting in symptoms that characterize a multitude of affective disorders^7^. Influential clinical models, for example, posit that little processing of the context in which a trauma took place makes resulting emotional memories prone to reactivation by trauma-related cues in safe contexts. This may manifest itself in symptoms that are characteristic of anxiety disorders and posttraumatic stress disorder (PTSD) such as fear generalization and intrusive images^8–12^. In support of this idea, experimental studies have demonstrated that reductions in contextual dependency of episodic memory indeed predict the occurrence or distress of analogue trauma intrusions^13–15^. Human fear-conditioning studies have similary shown that contextual information regulates recall of danger and safety^7,16,17^ (see Ref.^18^ for a calibration protocol that includes considerations about context) and contextual modulation of fear expression is impaired in PTSD^19^. Insights into how contextual dependency of emotional memory can be targeted is therefore of significant clinical interest.

The hippocampus—a brain region well-known for its crucial role in episodic memory^20^—serves several functions that could be capitalized on to foster contextual dependency of memory^7,21^. In humans, most of our understanding of these mechanisms comes from studies on neutral memories. This research has shown that a subregion of the hippocampus, the dentate gyrus, is involved in orthogonalizing a memory relative to similar memories, such that it becomes more distinct (i.e., pattern separation). This ensures that an episodic memory remains exclusively linked to its context and is not readily activated by a return to the context of a similar experience^22^. Other subregions, such as area CA1, are believed to aid in binding individual elements of a single experience, thereby making sure that context reinstatement accurately cues the respective memory^23^. Hence, proper integration of events in their encoding context involves increased discrimination in the dentate gyrus and stronger binding in the hippocampal CA1 area, together likely protecting against memory retrieval by resembling experiences. How can involvement of these regions be promoted such that contextual dependency of memory is *retrospectively* enhanced? Several fear-conditioning studies have identified a promising method to this end: reexposure to the spatial context in which encoding took place seems to neutralize later fear responses in other contexts^24–31^. Notably, brain regions involved in integration, such as hippocampal area CA1^26^, have been found to mediate such effects. It thus seems that integration of contextual information in existing emotional memory may facilitate the enhancement of contextual dependency by context reexposure.

Interestingly, interventions that target anxiety disorders and PTSD may partly work through this process, which underscores the importance of a detailed understanding of the effects of context reexposure on emotional memory. Indeed, contextual processing has been proposed as an important element of effective treatment^32,^ and interventions such as imaginal exposure^33^ and imagery rescripting^34^ involve the (imagined) revisiting of the trauma context (in addition to other crucial elements, e.g.^35^). A thorough understanding of these processes in relation to context reexposure may be imperative to ultimately improve treatment in clinical practice. For example, when executing in vivo exposure for anxiety disorders, a return to the same spatial context as during the emotional event is very often not possible (e.g., in cases of inaccessible or untraceable environments related to war traumas). Clinicians may, therefore, be inclined to expose their patients to contexts that perceptually resemble the encoding context as an alternative to reach comparable therapeutic effects. Similarly, when applying imagery techniques, a shallow imagination of the traumatic event may not lead to reinstatement of the encoding context but result in reactivation of a different or degraded context representation. Provided that memory integration is at play, it is doubtful whether desirable treatment outcomes would then be reached. This is because binding of the original emotional memory with a context that resembles the encoding context could be triggered, which may result in a more *generic*—instead of specific—memory representation. This could in turn generate *impaired*—not enhanced—contextual dependency of memory^25^. In line with this idea, rodent research suggests that when an animal is exposed to a context that is similar to the conditioning context, fear generalization is subsequently amplified^31,36^. Thus, exposure to a similar context could inadvertently be counterproductive. Knowing if and when such effects occur is critical to promote favorable treatment outcomes.

Insight into what factors drive these effects of context (re)exposure may be crucial to successfully target contextual dependency of memory. If integration of contextual information with an existing emotional memory underlies therapeutic effects, strong memory retrieval during context reexposure is probably required. This can be expected as previous research suggests that memory reactivation of an earlier event drives the integration of additional information^37–40^. Based on these insights, it thus seems likely that stronger retrieval during context reexposure predicts larger increases in contextual dependency. This may especially be true for emotional memories that typically depend less on context than neutral memories^4–6^. Stronger retrieval during exposure to a *similar* context may also amplify memory integration, but in this case of the original learning event with a different context^41^. Because integration of contextually dissimilar memories may reduce the specificity of memory^25^, stronger memory retrieval should then predict smaller contextual dependency.

**Figure 1:**
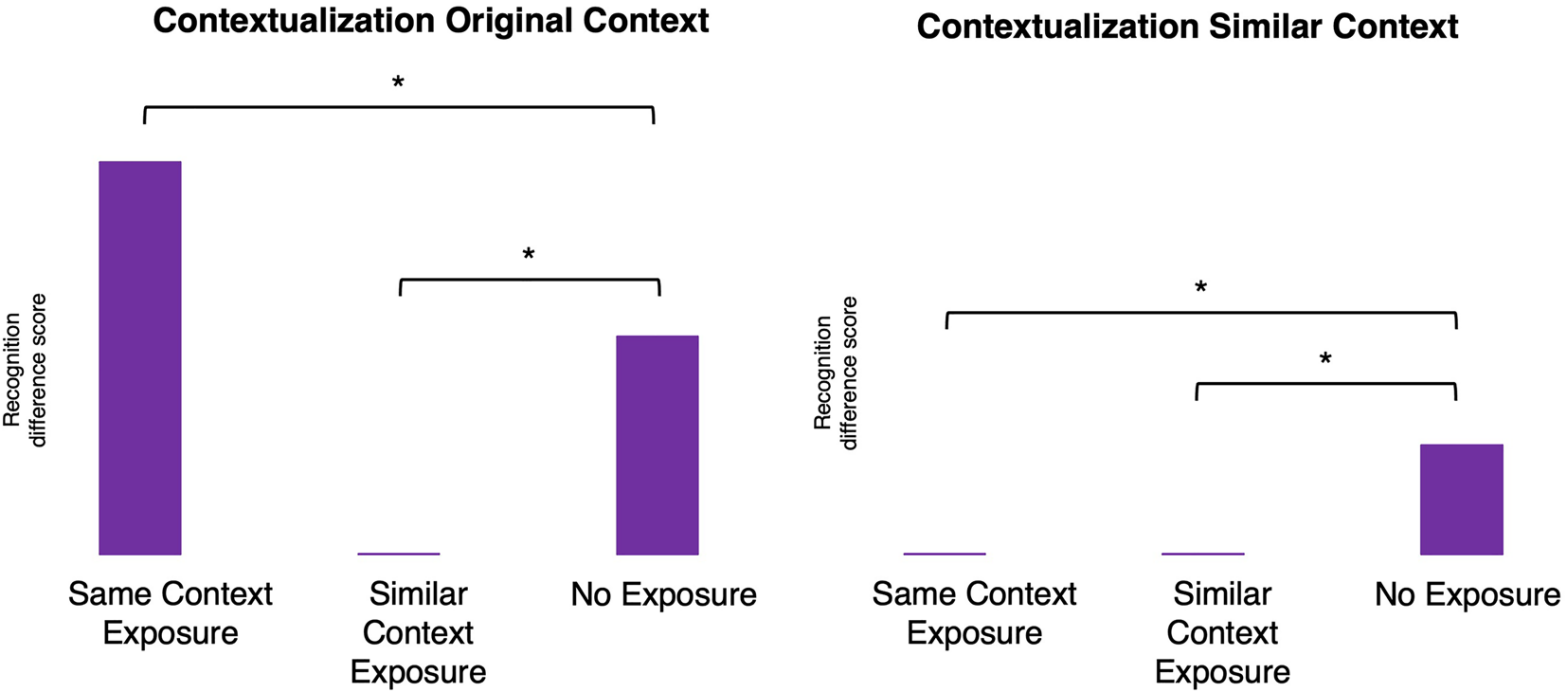
Illustration of predictions. An increase in contextual dependency of memory by context reexposure should be expressed as memory being highly specific for the original encoding context only. That is, the difference in performance between Test in Original Context and Test in New Context (Contextualization Original Context) would be enhanced relative to No Exposure, whereas the difference between Test in Similar Context and Test in New Context (Contextualization Similar Context) would be decreased. Impaired contextual dependency of memory by exposure to a similar context should be expressed as memory becoming more equal for testing in original, similar, and new contexts. Hence, the difference in performance between Test in Original Context and Test in New Context (Contextualization Original Context), as well as Test in Similar Context and Test in New Context (Contextualization Similar Context) should become relatively decreased compared to No Exposure.

In the present study, we thus aimed to test whether (i) reexposure to the encoding context improves contextual dependency of episodic memory while (ii) exposure to a similar context impairs it. Furthermore, we tested if alterations due to context (re)exposure (iii) become more pronounced with stronger memory retrieval during (re)exposure and (iv) whether these alterations are different in magnitude for emotional versus neutral memories. Since we were interested in the effects of context (re)exposure on consolidated memories (i.e., for which sleep is required^42^), we inserted a 24-h delay between encoding, context (re)exposure, and memory testing. On day 1, participants (*N* = 180) were shown a series of images of faces (neutral faces: *N* = 90, angry faces: *N* = 90) on background pictures (spatial contexts). One day later, the two groups were divided in three subgroups (*N* = 30) that were either (i) reexposed to the spatial contexts seen on day 1 (*Same Context Exposure group*), (ii) were shown similar contexts (*Similar Context Exposure group*), or (iii) did not come to the laboratory (*No Exposure group*). To stimulate memory retrieval and measure its strength, participants that were exposed to contexts (*Same Context Exposure group* or *Similar Context Exposure group*) were instructed to indicate to what extent they relived the episodes they had encoded on the first day. On the third and final day, all participants underwent a recognition test during which they were presented with targets (i.e., faces seen during encoding) and lures (i.e., unfamiliar faces). As a test of contextual dependency of memory^4–6^, one third of the targets and lures were presented on one of the contexts that were seen on the first day, i.e., the same context for targets and random contexts for lures (*Test in Original Context condition*). Another third of the faces was shown on a similar context (*Test in Similar Context condition*) and the final third was shown on a completely new context (*Test in New Context condition*). To assess contextual dependency as a function of context (re)exposure, we calculated two difference scores: Test in Original Context—Test in New Context (*Contextualization Original Context score*) and Test in Similar Context—Test in New Context (*Contextualization Similar Context score*). An increase in contextual dependency due to context reexposure should be expressed, relative to the No Exposure group, as an enhanced *Contextualization Original Context score*, together with a decreased *Contextualization Similar Context score* (Fig. 1). In other words, if contextual dependency is bolstered, memory should be highly specific for the original encoding context only. Impaired contextual dependency due to exposure to a similar context should, relative to the No Exposure group, be reflected as a decreased *Contextualization Original Context score*, together with a decreased *Contextualization Similar Context score*. Thus, if contextual dependency is impaired, memory should become roughly equal in original, similar, and new contexts. Furthermore, we expected that these effects would become more pronounced when memories were relived to a larger degree during context (re)exposure on day 2. Finally, as emotional memories should overall be characterized by relatively low contextual dependency, we predicted that the enhancing effect of context reexposure on contextual dependency would be larger, whereas the impairing effect of similar context exposure would be smaller, for emotional versus neutral memories.

## Materials and methods

### Participants

One hundred eighty subjects (138 females, 42 males) with a mean age of 21.94 years (*SD* = 2.83, range = 18–35) participated in the study. The participants were university students that were recruited using online (e.g., social media) and offline (e.g., flyers) advertisements. Informed consent was obtained from all these subjects. We excluded 18 participants who performed at chance level during the recognition test (i.e., in case of a higher than 5 percent chance that recognition responses were random, assessed by way of a binomial test as in previous studies^43^). In exchange for participation, the participants received either €24,—(*Same Context Exposure group* and *Similar Context Exposure group*) or €16,—(*No Exposure group*), or an equivalent number of course credit. All procedures were approved by the ethical committee of the Faculty of Social and Behavioral Sciences at Utrecht University (file #16–085). The study was performed in accordance with the relevant guidelines and regulations.

The participants were allocated to one of six groups. Half of these groups were shown angry faces in a spatial context (*Negative Face*, final *N* = 79), and the other three groups were shown neutral faces (*Neutral Face*, final *N* = 83). We included angry faces with gaze towards the subject, as this is recognized as a clear indication of threat directed at the subject (e.g., more so than fearful faces with direct gaze^44^). On the second day of the experiment, two of the groups were exposed to the same context as during encoding (*Negative Face—Same Context Exposure*, *N* = 26; *Neutral Face—Same Context Exposure*, *N* = 28), two other groups were exposed to similar contexts (*Negative Face—Similar Context Exposure*, *N* = 24; *Neutral Face—Similar Context Exposure*, *N* = 28), and the final two groups did not come to the laboratory in between encoding and testing (*Negative Face—No Exposure*, *N* = 29; *Neutral Face—No Exposure*, *N* = 27).

Based on the effect sizes in earlier research^4,5^, we expected that including 32 participants would be enough to detect context-dependent memory and smaller contextual dependency for emotional versus neutral memory (Cohen’s d = 0.5, 1 − β = 0.80, α = 0.05). However, since we added the Context (re)exposure factor, and in the present study the Emotion factor is manipulated between subjects, we tested 60 participants per group (Same Context Exposure, Similar Context Exposure, No Exposure). This approach also ensured that our sample would be large enough even after excluding participants who had performed at chance level. Additionally, we include Bayes factors to quantify evidence in favor of the null hypothesis (see Sect. "Data analysis").

### Stimuli

#### Faces

A total of 180 face images, drawn from the Radboud Faces Database^45^, the NimStim set of Facial Expressions^46^, and the Chicago Face Database (Version 2.0.3., July 2016)^47^, were included in our stimulus set. Ninety of these faces had a neutral expression (45 females, 45 males), and the other ninety had an angry expression (45 females, 45 males).

#### Contexts

As context images, we used a total of 180 pictures of indoor and outdoor spatial environments (e.g., a forest, a kitchen), like in previous research^4,5^. We selected two thirds of the images based on them resembling one of the other images in the set (e.g., two kitchens that looked similar to us). One third of the images were unique.

### Experimental task

#### Encoding

**Figure 2:**
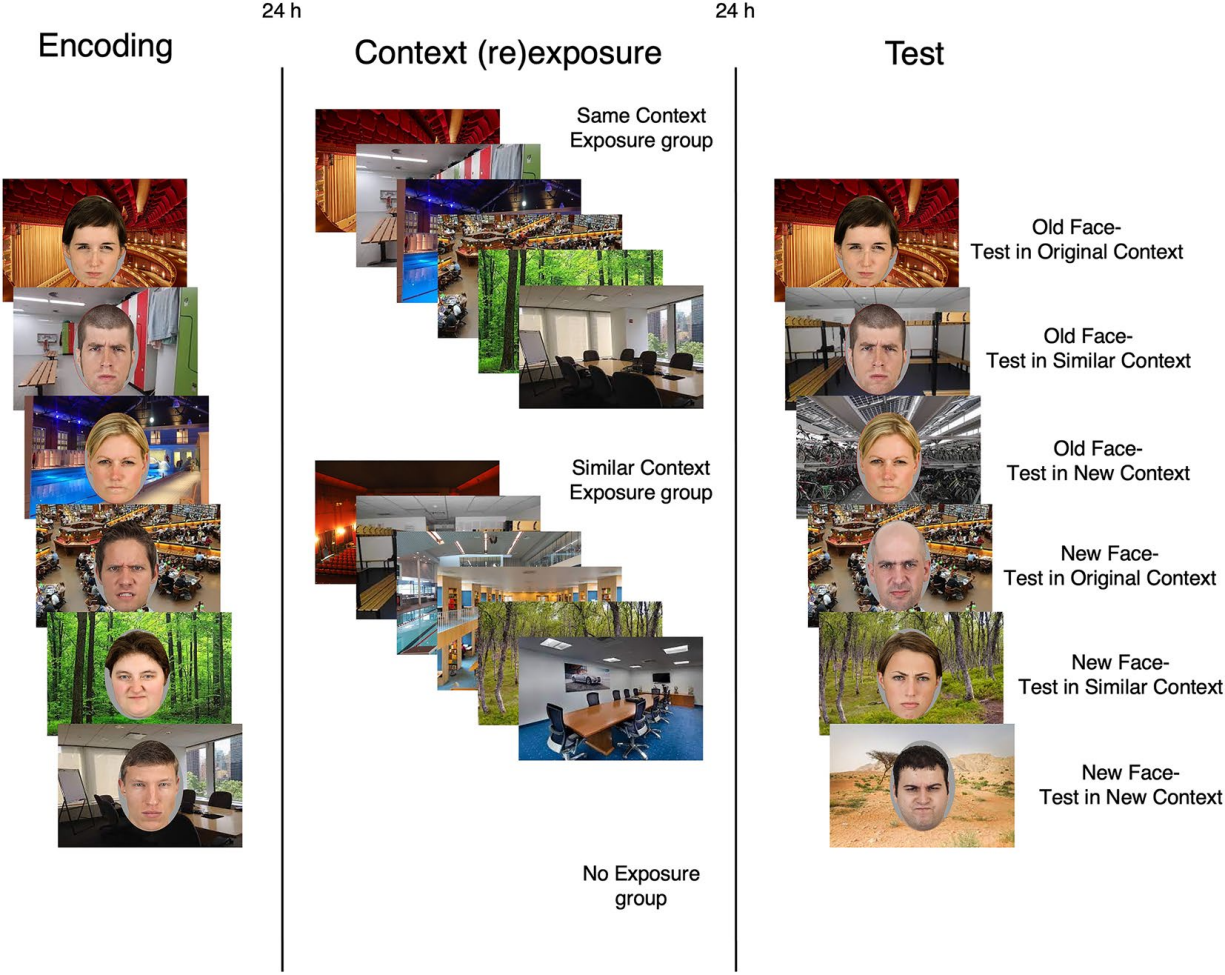
Experimental paradigm. Participants performed an encoding task, underwent context (re)exposure, and completed a recognition test on three consecutive days. During encoding, participants were randomly assigned to one of two groups and viewed either angry (Negative Face group, displayed here) or neutral faces (Neutral Face group, not displayed), presented on a context image. On day 2, the participants were again randomized, but now to one of three groups. That is, they were either reexposed to all the contexts they had seen on the previous day (Same Context Exposure group), were shown contexts similar to the ones they had seen before (Similar Context Exposure group) or did not come to the laboratory (No Exposure group). On the final day, all participants performed a recognition test, and were presented with faces they had seen on day 1 (Old Face) and unfamiliar faces (New Face). The faces were presented on a context they had seen during encoding (Test in Original Context), a similar context (Test in Similar Context), or a novel context (Test in New Context). The context images displayed in this figure illustrate those used in the experiment, which are not shown here due to copyright.

An overview of the experimental paradigm is displayed in Fig. 2. During encoding, participants were presented with 30 female and 30 male face images (angry in the *Negative Face group*, neutral in *the Neutral Face group*) on unique background pictures.

Each trial started with presentation of the background picture for 2 s. Next, the face was shown together with—and in the middle of—the background picture for 4 s. Then the face image disappeared, and only the background was shown for 0.5 additional seconds. Immediately afterwards, participants were asked to rate the vividness of their imagined scene on a visual analogue scale, ranging from “not vivid” to “very vivid”. Participants were given 2.5 s to respond, during which reaction times were recorded. The next trial started after the presentation of a black background (intertrial interval) for 1, 2, or 3 s (randomized in blocks of 3 trials). The participants completed three practice trials before the task commenced.

#### Context (re)exposure

During the context (re)exposure session, participants were either presented with all 60 background pictures they had seen during encoding (*Same Context Exposure group*) or shown 60 background picture that resembled the original background picture (*Similar Context Exposure group*). The *No Exposure group* did not come to the laboratory. For the *Same Context Exposure* and *Similar Context Exposure groups*, the context images were presented—without face images—for 3 s, during which participants rated reliving of their previously imagined scenes on a visual analogue scale, ranging from “no reliving” to “much reliving”. Reaction times were recorded. The next trial started after the presentation of a black background (intertrial interval) for 1, 2, or 3 s. The participants completed three practice trials before the task started.

#### Test

During the recognition test, participants were shown 30 of the 60 face images that were presented during encoding (i.e., 15 male and 15 female, randomly selected), and 30 new faces (15 male and 15 female, all angry in the *Negative Face group* and all neutral in the *Neutral Face group*) as lures. These faces were shown once (i.e., there was a total of 60 trials in the recognition task). To assess contextual dependency of the memories, 10 of the old face images were presented on the original encoding context of day 1 (*Test in Original Context*), another 10 on a context similar to the encoding context (*Test in Similar Context*), and the final 10 on a context that participants had not seen before (*Test in New Context*). Likewise, the lures were either shown on a context seen on day 1 (images that were not used for recognition of old faces in original contexts), a similar context (images that were not used for recognition of old faces in similar contexts), or a new context (10 new images).

For each trial, the context image was presented first, for 3 s. Then, the face image appeared during which participants could indicate whether they had seen the face before or not (yes/no). After participants had pressed yes (old) or no (new), they were asked to indicate the level of confidence in their response. If they had responded “no”, they were shown three response options to indicate how sure they were that the face was new (1 = sure, 2 = probably, 3 = guess). When participants had responded “yes”, they were asked to indicate their level of confidence that the face was old (4 = guess, 5 = probably, 6 = sure). Both the yes/no recognition as well as the confidence rating task were self-paced, and response times were recorded. The next trial started after presentation of a black background (intertrial interval) for 1, 2, or 3 s. Participants performed three practice trial before the task commenced.

#### Trial order

For each of the three phases (encoding, context (re)exposure, test), trials were presented in blocks of twelve, in which trials that correspond to each within-subjects condition were equally distributed (e.g., *Test in Original Context*, *Test in Similar Context*, *Test in New Context* for old and new faces). As such, trials from each within-subjects condition were not presented more than three times successively. The order of blocks was the same across the encoding, context (re)exposure, and test phases, but the order of trials within blocks was randomized for each phase.

### Procedure

Upon arrival on day 1, participants were asked to read an information brochure which stated that they would be viewing face images on background pictures, use their imagination during the experiment, and answer questions about the presented images. We purposely kept participants unaware that they would undergo memory tests to prevent them from actively rehearsing the learned material between sessions. After they had given written consent, participants were asked to complete questionnaires (State and Trait Anxiety Inventory^48^ and Beck Depression Inventory- II^49^). These were collected for potential exploratory purposes, but not further analyzed.

#### Encoding

Participants were informed that they would be shown background pictures on a computer screen in which a face image would appear after some time. They were instructed to imagine as vividly as possible a scene that involves the face image in the background. It was explained that after the face image and background pictures had disappeared from the screen, they could rate the vividness of their imagined scene within approximately two seconds, using a continuous scale.

#### Context (re)exposure

Twenty-four hours later, participants returned to the lab for a context (re)exposure session. Participants allocated to the *Same Context Exposure* or *Similar Context Exposure groups* were explained that they would be shown background images and were instructed to rate within three seconds how vividly they remembered a scene they had imaged on day 1 in relation to the presented image. Participants in the *No Exposure group* did not come to the lab and thus were not exposed to any of the previously presented contexts.

#### Test

On the third and final day, participants were asked to complete a recognition test of the faces they had seen on day 1. It was explained that they would be presented with background pictures and face images, and that some faces had been presented during the encoding session on day 1 while others had not been shown before. They were instructed to respond whether they recognized the face as one they had seen on day 1 (old) or not (new). It was explained that after participants had pressed “old” or “new”, they would rate their level of confidence in this response (i.e., 1 = very sure new, 2 = somewhat sure new, or 3 = guess new if they had responded “new”, and 4 = guess old, 5 = somewhat sure old, or 6 = very sure old if they had responded “old”).

After the recognition task, participants were asked to complete a short questionnaire concerning encoding characteristics (e.g., how well they could focus during the task) and overall motivation for each part of the experiment. Finally, they were debriefed and paid for their participation.

### Data analysis

We performed both Frequentist and Bayesian analyses. For the Frequentist approach, we used The Statistical Package for the Social Sciences (SPSS) version 28.0.1.0 (Armonk, NY: IBM Corp.) in case of analyses on the subject level (see Sects. "Vividness", "Reliving" and "Recognition"). For analyses on the level of trials (see Sect. "Interactions between reliving strength and changes in contextual dependency by context (re)exposure"), we used R (lme4 and LmerTest packages in version 4.0.3). Two-sided tests were performed, and alpha was set at 0.05 for all these tests.

Bayesian analyses were executed with JASP version 0.17.2.1^50^, using default priors. For ANOVAs, we report Bayes inclusion factors (BF_incl_), which reflect evidence for inclusion of the factor, relative to all models excluding that factor. For t-tests, we report BF_10_, which reflects evidence for the alternative hypothesis. Regarding interpretation of these Bayes factors, note that values higher than 10 have been classified as strong evidence, values between 3 and 10 as moderate evidence, and values between 3 and 1 as anecdotal evidence for factor inclusion (BF_incl_) or the alternative hypothesis (BF_10_). Bayes factors lower than 1/10, between 1/3 and 1/10, and between 1/3 and 1 have been classified as strong, moderate, and anecdotal evidence, respectively, for factor exclusion (in case of BF_incl_) or the null model (in case of BF_10_)^51^. We performed assessments of normality (Shapiro–Wilk) and homogeneity of variance (Levene’ test and variance ratios) for all analyses (although for repeated measures ANOVAs we checked sphericity instead of variance, using Mauchly’ test). Both assumptions were met, unless reported otherwise in the Sect. "Results".

#### Vividness

##### Pre-processing

We calculated average vividness scores for all six groups. We included only 50 of the total 60 vividness trials in our analysis because 10 face-context combinations were not related to any test condition on day 3. That is, *Old Face—Test in Original Context*, *Old Face—Test in Similar Context*, *Old Face—Test in New Context*, *New Face—Test in Original Context*, and *New Face—Test in Similar Context* all include a face image or context image that were presented on day 1, which leaves 10 trials that are not relevant for performance on day 3. Participants who did not fill in any vividness rating in time (i.e., within 2.5 s) were excluded (*Negative Face—Similar Context Exposure*, *N* = 1; *Negative Face—No Exposure*, *N* = 3; *Neutral Face—Same Context Exposure*, *N* = 2; *Neutral Face—No Exposure*, *N* = 1).

##### Statistical analyses

To check whether the context (re)exposure groups did not significantly differ in vividness of the imagined scenes on day 1 and explore whether vividness of scenes that included angry versus neutral faces differed, we performed a two-way ANOVA. We tested a main effect of Emotion (*Negative Face* versus *Neutral Face*), a main effect of Context (Re)exposure (*Same Context Exposure* versus *Similar Context Exposure* versus *No Exposure*), and an Emotion × Context (Re)exposure interaction. Tukey’s HSD (honestly significant difference) tests were performed in case of significant main or interaction effects.

#### Reliving

##### Pre-processing

Reliving scores were calculated for the *Negative Face—Same Context Exposure*, *Negative Face—Similar Context Exposure*, *Neutral Face—Same Context Exposure*, and *Neutral Face—Similar Context Exposure groups*. Again, we included 50 of the 60 trials, as only those were relevant for the memory tests performed on day 3 (see Sect. "Vividness Pre-processing"). Participants who did not fill in any reliving rating in time (i.e., within 3 s) were excluded (*Negative Face—Similar Context Exposure*, *N* = 1; *Neutral Face—Same Context Exposure*, *N* = 2).

##### Statistical analyses

To assess whether reliving of the scenes that were imaged on day 1 was higher when the same versus a similar context was presented on day 2 and explore whether this effect depends on whether the day 1 memories were emotional, we performed a two-way ANOVA. In this analysis, main effects of Emotion (*Negative Face *versus *Neutral Face*), and Context (Re)exposure (*Same Context Exposure* versus *Similar Context Exposure*), as well as an Emotion × Context (Re)exposure interaction were included.

#### Recognition

##### Pre-processing

We calculated hit and false alarm rates for all three within-subject conditions (*Test in Original Context*, *Similar Context*, or *New Context*) in all 6 groups. From these hits and false alarms data, we calculated d-prime sensitivity index. To do so, hit rates higher than 0.95, and false alarm rates smaller than 0.05 were truncated at 0.95 and 0.05, respectively^52^.

Subsequently, we calculated two difference scores (*Test in Original Context—Test in New Context*, and *Test in Similar Context—Test in New Context*). These constitute the variables of interest to assess changes in contextual dependency of memory (*Contextualization Original Context* and *Contextualization Similar Context*, respectively).

##### Statistical analyses

Manipulation check: contextual dependency of emotional and neutral memories. To assess whether correct recognition depended on context, and this contextual dependency was lower for emotional than neutral memories, we analyzed d-prime scores in the *No Exposure control group*. We only included the control group for this analysis, because in earlier studies that we aimed to replicate here no exposure to context was included between encoding and testing^4–6^. To test if contextual dependency of emotional memories is impaired relative to neutral memories, we performed two independent t-tests. The independent variable was Emotion (*Negative Face* versus *Neutral Face*), and dependent variables were d-prime difference scores (*Contextualization Original Context*, and *Contextualization Similar Context*). If contextual dependency of emotional memory is relatively low, then the *Contextualization Original Context* and *Contextualization Similar Context scores* should both be smaller in the *Negative Face* than the *Neutral Face group*. If these effects were indeed observed, we analyzed contextual dependency of emotional and neutral memories separately by way of repeated measures ANOVAs with Test (*Test in Original Context*, *Test in Similar Context*, *Test in New Context*) as the independent variable, and the d-prime score as the dependent variable. Planned comparisons were performed to compare accuracies between the conditions if an effect of Test was observed. When no relatively low contextual dependency of emotional memory was found, we collapsed the data of the *Negative Face* and *Neutral Face conditions*.

Hypothesis test: effects of context (re)exposure on contextual dependency. To assess whether context reexposure modulates contextual dependency of memory, we performed univariate ANOVAs with Emotion (*Negative Face*, *Neutral Face*) and Context (Re)exposure (*Same Context Exposure*, *Similar Context Exposure*, *No Exposure*) as independent variables and the d-prime difference scores (*Contextualization Original Context*, and *Contextualization Similar Context*) as dependent variables. If no Emotion × Context (Re)exposure interaction was found, we collapsed the data of the *Negative Face* and *Neutral Face groups* to test for an effect of Context (Re)exposure on contextual dependency. In case of significant effects, we performed planned contrasts to see if (i) the *Contextualization Original Context score* is higher, but the *Contextualization Similar Context score* is lower in the *Same Context Exposure group* versus the *No Exposure group* (i.e., greater contextual dependency, see Fig. 1), whereas (ii) both the *Contextualization Original Context* and *Contextualization Similar Context scores* are lower in the *Similar Context Exposure group* versus the *No Exposure group* (i.e., smaller contextual dependency).

#### Interactions between reliving strength and changes in contextual dependency by context (re)exposure

##### Pre-processing

To regress possible changes in contextual dependency on retrieval strength, we linked the recognition responses for old and new faces on day 3 with the respective reliving scores (0–100) during context exposure on day 2 on the level of trials for 5 within-subject conditions (*Old Face—Test in Original Context*, *Similar Context*, or *New Context*, *New Face*—*Test in Original Context* or *Similar Context*). Note that the new faces (lures) were of course themselves never presented during context (re)exposure, but the contexts they were presented on during testing were in fact shown in this phase. However, new faces presented on new contexts on day 3 are (by definition) not related to any reliving scores on day 2, such that no corresponding reliving score exists for *New Face—Test in New Context*. This absence of a relationship between recognition response and retrieval strength needs to be included in the model to predict d-prime accuracy in new contexts that are based on recognition of old faces. Since 10 of the 60 trials on day 2 (context (re)exposure) are not related to any test trial on day 3 (see Sects. "Vividness Pre-processing" and "Reliving Pre-processing"), these reliving trials can be used to create this absence of a relationship between recognition responses and reliving for *New Face—Test in New Context*. We randomly paired the reliving scores of these 10 remaining trials on day 2 with recognition responses during presentation of new faces on new contexts on day 3. Due to this random pairing, no relationship should emerge such that the *Test in New Context condition* can be included in the model as reference condition.

##### Statistical analyses

To assess if contextual dependency (i) becomes larger in the *Same Context Exposure group*, but (ii) smaller in the *Similar Context Exposure group*, with stronger memory retrieval during context (re)exposure, we performed a multilevel probit regression. The trial level coefficients yielded by this type of model are Z-scores and have been shown to be mathematically equivalent to d-prime^53^, the key measure from signal detection theory that is typically computed by aggregating data within participants. Therefore, their interpretation is the same as d-prime: the beta parameters of this regression represent an increase or decrease in the probability of a ‘yes’ response associated with each variable. As fixed effects on the item level (1st level), we entered Reliving (0–100, group-mean centered), Face (*Old*, *New*), and Test (*Test in Original Context*, *Test in Similar Context*, *Test in New Context*). On the participant level (2^nd^ level) we entered Emotion (*Negative Face*, *Neutral Face*). The *Same Context Exposure* and *Similar Context Exposure groups* were analyzed separately. Random intercepts were included for participants^54^.

First, we aimed to assess whether the random pairing of reliving scores with recognition responses for *New Face—Test in New Context* was successful and thus appropriate to use as reference (see Sect. "Interactions between reliving strength and changes in contextual dependency by context (re)exposure Pre-processing"): here no significant relationship should occur between reliving strength and memory accuracy. For the role of reliving strength during context (re)exposure in later contextual dependency of memory, we expected that higher reliving in the *Same Context Exposure group* would predict (i) improved memory for *Test in Original Context* versus *Test in New Context* (i.e., *Contextualization Original Context*) and (ii) reduced memory for *Test in Similar Context* versus *Test in New Context* (i.e., *Contextualization Similar Context*). Furthermore, we expected that higher reliving in the *Similar Context Exposure group* would predict reduced memory for both (iii) *Test in Original Context* versus *Test in New Context* (i.e., *Contextualization Original Context*) and (iv) *Test in Similar Context* versus *Test in New Context* (i.e., *Contextualization Similar Context*). We first tested an interaction with Emotion for each of these effects (i.e., Emotion × Reliving × Test × Face). If no significant 4-way interactions were observed, we collapsed the data of the *Negative Face* and *Neutral Face groups*. We then tested Reliving × Test (*Test in Original Context* versus *Test in New Context*) × Face (*Old* versus *New*) and Reliving × Test (*Test in Similar Context* versus *Test in New Context*) × Face (*Old* versus *New*) interactions for the *Same Context Exposure group* and the *Similar Context Exposure group* separately. Planned comparisons were performed in case of a significant effect to assess the predicted relationships between reliving strength and the test conditions.

## Results

### Manipulation checks

#### Vividness

**Figure 3:**
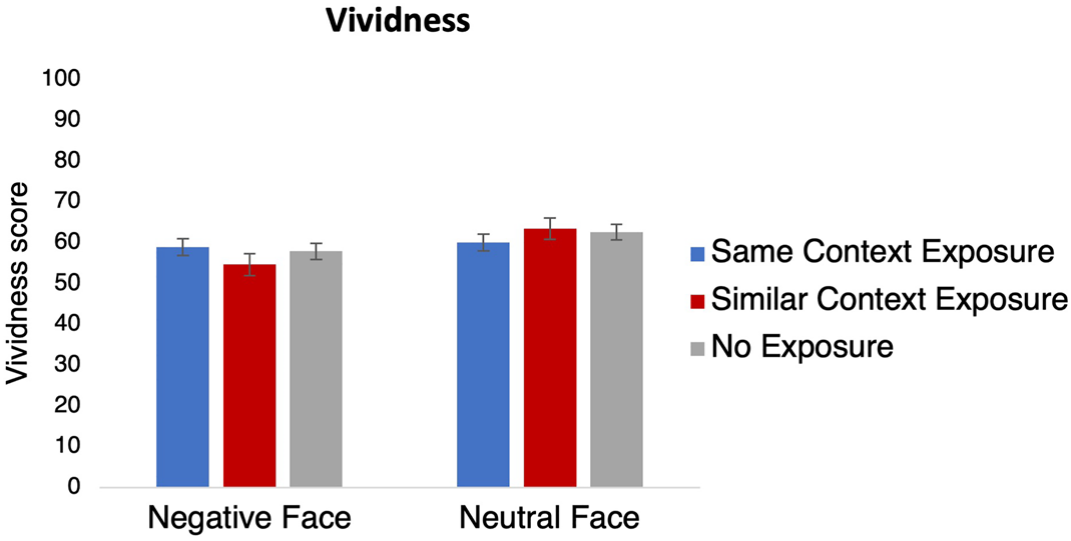
Vividness ratings during the encoding task in the Same Context Exposure (blue bars), Similar Context Exposure (red bars), and No Exposure group (grey bars). The scores are plotted separately for the Negative Face (left) and Neutral Face (right) groups. Error bars represent SEM.

Vividness of the imagined scenes during encoding was lower for negative than neutral memories (Emotion, *F*_1,150_ = 7.08, *p* = 0.01, *η*_*p*_^*2*^ = 0.05, BF_incl_ = 4.04, Fig. 3). Crucially, there were no significant differences in vividness between the *Same Context Exposure*, *Similar Context Exposure*, and *No Exposure groups* (Context (Re)exposure, *F*_2,150_ = 0.14, *p* = 0.87, *η*_*p*_^*2*^ = 0.002, BF_incl_ = 0.07). We also did not observe an interaction between these two factors (Emotion × Context (Re)exposure, *F*_2,150_ = 1.44, *p* = 0.24, *η*_*p*_^*2*^ = 0.02, BF_incl_ = 0.34). These findings thus confirm that no unexpected differences in vividness occurred between the groups. Note that usually higher vividness scores are found for emotional than neutral memories^55^. However, these previous studies have mostly focused on vividness during recall of a previously formed memory, not during imagination in an encoding task as we did here (but see Ref.^56^). The relatively low vividness score for emotional memories is consistent with a previous study in which a similar paradigm was used and may reflect decreased unitization of separate items in memory^57^.

For one of the six groups (Angry Face—Same Context Exposure), Shapiro–wilk showed a significant deviation from normally distributed data (*W* = 0.861, *p* = 0.002). According to central limit theorem, distributions that include sample sizes higher than 30, which applies to the analyses of main effects here, can be regarded as normal. Note also that ANOVAs are generally believed to be quite robust to limited violations of the normality assumption^58^.

#### Reliving

**Figure 4:**
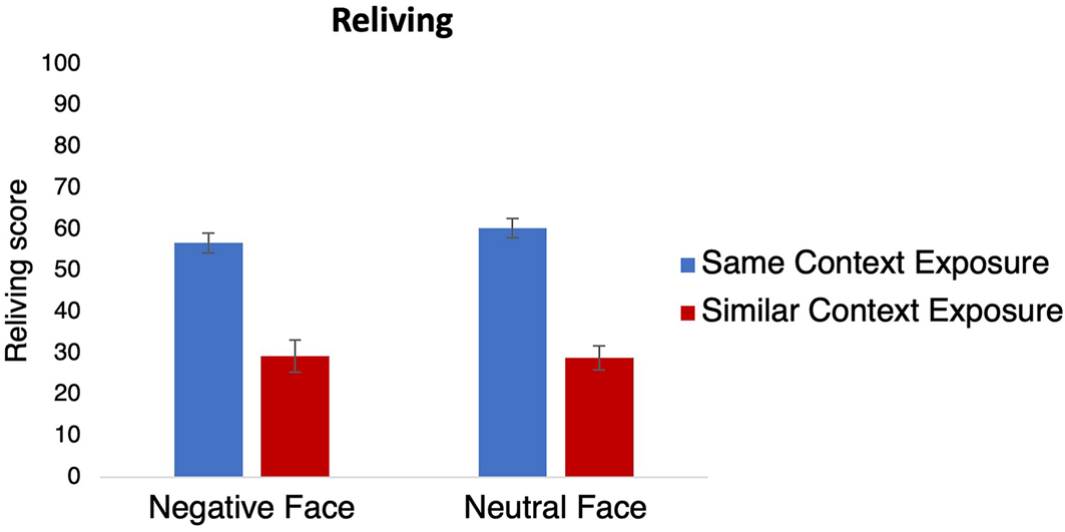
Reliving ratings in the Same Context Exposure (blue bars) and Similar Context Exposure (red bars) group. The scores are plotted separately for the Negative Face (left) and Neutral Face (right) groups. Error bars represent SEM.

Analyses of reliving ratings on day 2 confirmed that context reexposure led to more reliving than exposure to a similar context (Context (Re)exposure, *F*_1,99_ = 97.01, *p* < 0.001, *η*_*p*_^*2*^ = 0.50, BF_incl_ = 3.25 × 10^13, Fig. 4). This effect did not depend on whether memories were negative or neutral (Emotion × Context (Re)exposure, *F*_1,99_ = 0.13, *p* = 0.72, *η*_*p*_^*2*^ = 0.001, BF_incl_ = 0.30). Finally, there was no overall difference in reliving for negative versus neutral memories (Emotion, *F*_1,99_ = 0.52, *p* = 0.47, *η*_*p*_^*2*^ = 0.01, BF_incl_ = 0.25). In short, inducing relatively strong reliving by reexposure to the encoding context was successful, and no other unexpected effects on reliving occurred.

Levene’s test of equality of error variances was significant (*F*_3, 99_ = 3.05, *p* = 0.032). Yet, the maximum variance ratio was relatively low (2,75, see Ref.^59^ for comparison), indicating that high sample size may have partly caused the significant result. Note also that ANOVAs are believed to be quite robust to small deviations from equal error variances^58^.

#### Contextual dependency of emotional and neutral memories

We observed that, for the *No Exposure groups*, the *Contextualization Original Context score* (*t*_54_ = 1.78, *p* = 0.08, *d* = 0.48, BF_10_ = 0.99) and the *Contextualization Similar Context* score (*t*_54_ = 2.09, *p* = 0.04, *d* = 0.56, BF_10_ = 1.62) were at least trend significantly smaller in the *Negative Face group* than the *Neutral Face group* (Fig. S1). These findings thus suggest that contextual dependency was lower for emotional than neutral memories. Furthermore, for neutral memories, recognition accuracy was dependent on context (Test, *F*_2,52_ = 9.83, *p* < 0.001, *η*_*p*_^*2*^ = 0.27, BF_incl_ = 229.59, Fig. S2B, right pane). Planned comparisons suggested that accuracy scores were highest in the original encoding context, lower in the similar context, and lowest in the new context (*Test in Original Context* versus *Test in Similar Context, F*_1,26_ = 6.61, *p* = 0.02, *η*_*p*_^*2*^ = 0.20, BF^10^ = 3.12; *Test in Similar Context* versus *Test in New Context*, *F*_1,26_ = 3.65, *p* = 0.07, *η*_*p*_^*2*^ = 0.12, BF^10^ = 0.99). Emotional memories also depended on context (Test, *F*_2,56_ = 3.67, *p* = 0.03, *η*_*p*_^*2*^ = 0.12, BF^10^ = 2.31, Fig. S2A, right pane), but here recognition was only elevated in the original versus similar context (*Test in Original Context* versus *Test in Similar Context*,* F*_1,28_ = 8.50, *p* = 0.01, *η*_*p*_^*2*^ = 0.23, BF^10^ = 6.23; *Test in Similar Context* versus *Test in New Context*, *F*_1,28_ = 1.04, *p* = 0.32, *η*_*p*_^*2*^ = 0.04, BF^10^ = 0.32). In short, these results largely replicate earlier studies showing context-dependent memory^1,2,4–6^, and smaller contextual dependency for emotional versus neutral memories^4–6^.

### Hypothesis tests

#### Effects of context (re)exposure on contextual dependency

**Figure 5:**
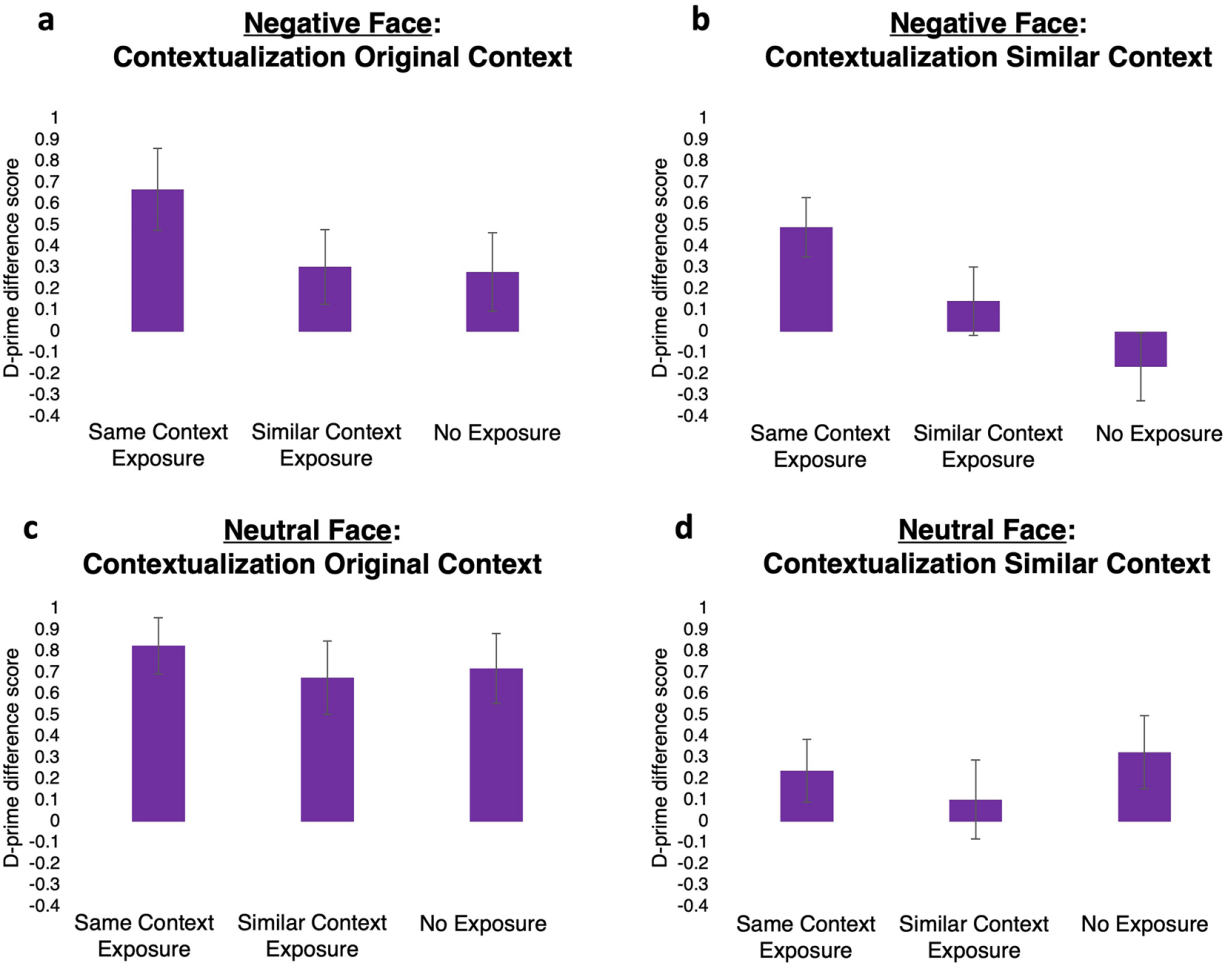
Contextualization Original Context (**a**) and Contextualization Similar Context scores (**b**) in the Negative Face group. Contextualization Original Context (**c**) and Contextualization Similar Context scores (**d**) in the Neutral Face groups Error bars represent SEM.

Whether memories were emotional or neutral did not significantly influence any effects of context (re)exposure on contextual dependency (Emotion × Context (Re)exposure, *F*_2,156_ = 0.38, *p* = 0.69, *η*_*p*_^*2*^ = 0.005, BF_incl_ = 0.15 for *Contextualization Original Context*; Emotion × Context (Re)exposure, *F*_2,156_ = 2.84, *p* = 0.06, *η*_*p*_^*2*^ = 0.035, BF_incl_ = 1.09 for *Contextualization Similar Context*). Therefore, we collapsed the data of the *Negative Face* and *Neutral Face groups*. Crucially, and contrary to expectations, contextual dependency did not differ between the context (re)exposure groups in the collapsed data (*F*_2,156_ = 1.46,* p* = 0.24, *η*_*p*_^*2*^ = 0.02, BF_incl_ = 0.21 for *Contextualization Original Context* and *F*_2,156_ = 1.80, *p* = 0.17, *η*_*p*_^*2*^ = 0.02, BF_incl_ = 0.28 for *Contextualization Similar Context*, see Figs. 5, S2, S3 and S4 for more detail).

#### Interactions between reliving strength and changes in contextual dependency by context (re)exposure

We first assessed whether the impact of reliving in the reference condition was indeed absent. That is, we tested whether including new faces presented in new contexts in the model related to reliving of the randomly paired contexts. Here, we found no main effect of reliving nor an interaction between Reliving and Emotion in either the *Same Context Exposure* or the *Similar Context Exposure group* (*Same Context Exposure group*: Reliving, *Z* = − 0.05, *p* = 0.623, Reliving × Emotion (*Negative* versus *Neutral*), *Z* = 0.02, *p* = 0.895; *Similar Context Exposure group*: Reliving, *Z* = 0.02, *p* = 0.786, Reliving × Emotion (*Negative versus Neutral*), *Z* = -0.09, *p* = 0.527). This shows that random pairing of reliving scores with recognition responses was successful and *Test in New Context* can be used as the reference condition in the models (see Sects. "Interactions between reliving strength and changes in contextual dependency by context (re)exposure Pre-processing" and "Interactions between reliving strength and changes in contextual dependency by context (re)exposure Statistical analyses"). Whether memories were negative or neutral did not modulate any interactions between reliving strength and contextual dependency (*Same Context Exposure group*: Emotion (*Negative* versus *Neutral*) × Reliving × Test (*Test in Original Context* versus *Test in New Context*) × Face (*Old* versus *New*), *Z* = 0.12, *p* = 0.639, Emotion (*Negative* versus *Neutral*) × Reliving × Test (*Test in Similar Context* versus *Test in New Context*) × Face (*Old* versus *New*), *Z* = − 0.23, *p* = 0.366; *Similar Context Exposure group*: Emotion (*Negative* versus *Neutral*) × Reliving × Test (*Test in Original Context* versus *Test in New Context*) × Face (*Old* versus *New*), *Z* = − 0.06, *p* = 0.810, Emotion (*Negative* versus *Neutral*) × Reliving × Test (*Test in Similar Context* versus *Test in New Context*) × Face (*Old* versus *New*), *Z* = 0.21, *p* = 0.425). Therefore, we again collapsed the data of the *Negative Face* and *Neutral Face groups*. Crucially, and in contrast with the predictions, reliving did not relate to the strength of contextual dependency in either the *Same Context Exposure group* (Reliving × Test (*Test in Original Context* versus *Test in New Context*) × Face (*Old* versus *New*), *Z* = − 0.05, *p* = 0.701; Reliving × Test (*Test in Similar Context* versus *Test in New Context*) × Face (*Old* versus *New*), *Z* = − 0.05, *p* = 0.700), or the *Similar Context Exposure group* (Reliving × Test (*Test in Original Context* versus *Test in New Context*) × Face (*Old* versus *New*), *Z* = 0.00, *p* = 0.979; Reliving × Test (*Test in Similar Context* versus*Test in New Context*) × Face (*Old* versus *New*), *Z* = 0.09, *p* = 0.510).

## Discussion

In the present study, we tested whether contextual binding of human episodic memory can be changed by (re)exposure to contexts that are perceptually identical or that resemble the original encoding context. We also assessed whether these effects are predicted by the strength of memory retrieval during (re)exposure and differ in magnitude for emotional versus neutral memories. We found that correct recognition was dependent on test context, and that this contextual dependency seemed lower for emotional than neutral memories. We also observed that memory retrieval was stronger during reexposure to the original encoding context than during exposure to a similar context. These findings replicate earlier studies^4–6^, and show that our basic manipulations were successful. However, we did not find evidence for any of the hypotheses: reexposure to the original encoding context did not promote contextual dependency of memory, nor did exposure to a similar context reduce it. Relevant Bayes factors showed moderate evidence in favor of the null hypothesis, thus indicating that a lack of statistical power does not explain these results. Also, no relationships were observed between retrieval strength during (re)exposure to the contexts and contextual dependency at test. Finally, no differences were found between emotional and neutral memories in any of these hypothesized effects. Therefore, the findings of this large-scale study do not support the idea that context (re)exposure after a neutral or emotional event modulates contextual dependency of memory.

These observations clearly contrast with fear-conditioning studies that did show effects of context (re)exposure on fear generalization in animals^24,26–31^ and humans^25^. One obvious difference between these previous studies and the present experiment is the emotional intensity of the memories. Whereas fear conditioning involves the administration of—sometimes severe—electric shocks, we here presented threatening faces, which in all likelihood are experienced as only mildly fearsome. It has been suggested that impaired contextual processing of emotional memories, by amygdalar downregulation of the hippocampus, may only occur under particularly strong conditioning protocols^60^, which are more aversive than the kind of procedures adopted here^61^. Similarly, it is well-known that generalization of fear to stimuli that are not predictive of threat is more common with high US intensities in fear-conditioning research^62^. One could thus suspect that the present design was not as well suited to investigate changes in contextual dependency of emotional memories as fear-conditioning paradigms. However, it is important to note that we did observe the expected smaller contextual dependency of emotional versus neutral memories like in previous studies^4–6^ (although one of the comparisons did not reach significance and the relevant Bayes factors showed anecdotal evidence at best). Therefore, it seems that the threatening faces were arousing enough to at least partly impair the processing of context, such that the premise to study changes in contextual binding of emotional events was met.

Conversely, instead of suspecting that the memories in this study were not emotionally intense enough, one may also wonder if the induced memories were in fact too strong to be changeable. Indeed, previous research has shown that particularly strong memories are relatively insensitive to change upon memory retrieval^63^. The d-prime scores in this study, which reflect memory performance and hence can be regarded as a proxy for overall memory strength, were not higher than 2 in any condition. Given that the highest possible d-prime score, using the formula we adopted here (including truncation) is 3.29, we can at least conclude that performance did not approach ceiling level. Also, previous research that provided evidence for changes in contextual dependency by e.g., administration of cortisol^4^ showed higher overall d-prime scores, suggesting that the memories in the present study were probably not of such high strength that they were generally insensitive to manipulation. Finally, if memory strength would be a crucial factor, the degree of reliving during context reexposure (which arguably depends on memory strength), could have been related to changes in contextual dependency by context (re)exposure, which is something the multilevel probit regression did not provide evidence for. Therefore, we believe that other factors probably better explain the null findings.

Considering the multilevel probit regression, one could also wonder whether the utility of this analysis was limited by the stimulus material that was included in this study. We have selected images of similar contexts (e.g., two kitchens) ourselves. If the degree of similarity (e.g., not too high) perhaps determines the effects of similar-context exposure on contextual dependency, then it would be possible that our subjective judgements may not have led to the level of similarity that is needed for the occurrence of the predicted effects. However, when we collected the stimulus material, we purposely selected images that – in our view – varied in their degree of similarity, so that reliving ratings on day 2 would show considerable spread. Additional calculations show that the average standard deviation in reliving ratings was 25.33 within participants. Furthermore, 75% of the participants in the Similar Context Exposure group provided at least one reliving rating lower than 20 and at least one rating higher than 80 (scale 0–100). Based on this spread and the finding that reliving in the Similar Context Exposure group was significantly lower than in the Same Context Exposure group (and still above zero), we believe that the presentation of similar contexts that very in their degree of similarity seems successful. We still did not find any effects, including for same context exposure, where issues in terms of contextual similarity by definition do not apply. Therefore, the idea that the degree of similarity between contexts may have hampered accurate tests of some of our predictions overall does not seem to be a convincing explanation for the null findings.

One major difference between previous relevant studies^24–31^ and the present experiment is the outcome variable: we tested for contextual dependency of declarative memory instead of conditioned fear responses. The psychophysiological indices in fear-conditioning research by no means perfectly reflect clinical anxiety^64^, yet give an indication of whether context (re)exposure causes changes in fear, which is something declarative-memory paradigms do not allow. Having the possibility to check whether contextual dependency was smaller for emotional than neutral memories was one of the reasons we adopted this declarative memory paradigm. This method also allowed for the inclusion of many trials per participant, which enabled us to assess relationships between memory reliving during context (re)exposure and contextual dependency at test in a statistically powerful manner. Even though it is not known how episodic memory contextualization maps onto fear generalization, it has been proposed that contextual processing deficits may subserve each (e.g.,^7^). Likewise, broad impairment in hippocampus-dependent associative learning has been revealed as a vulnerability factor for PTSD^11^, and associative learning of foreground cues and background context has been proposed as an essential ingredient to form an integrated representation of an event^11^. Thus, at least in theory, one would suspect that contextual dependency of declarative memory and conditioned fear responses could be affected by context (re)exposure in similar ways.

## Limitations and future directions

It must be noted that there are several disadvantages to the paradigm we used here, which may have prevented us from observing the hypothesized effects of context (re)exposure. In animal fear-conditioning research, rodents typically explore a context for several minutes during fear conditioning (e.g.,^27^). The extensive exploration of the context, together with the occurrence of a threatening shock, leads to the formation of a robust contextual memory. This firmly established memory can then be updated by reexploration, through the integration of contextual information that the animal had not yet learned or had already forgotten^26^. In the paradigm adopted here, initial learning involved the imagination of many scenes based on faces of similar expressions (either all angry or all neutral) in contexts, which were presented in quick succession. Conceivably, this method made it somewhat difficult for participants to imagine clear and well-defined episodes. As episodic memory has traditionally been viewed as memory for specific events that include what-where-when qualities which elicit a recollective experience during recall^65^, it is somewhat doubtful that we induced the formation of complete and truly episodic memories. Words—instead of faces—in contexts, like we used in recent research^40^, may be more effective to do so. Without clearly established episodic memories, it is possible that during context (re)exposure participants did not learn information to integrate with the existing memory. Apart from difficulties in the formation of unique episodic memories during learning, the relatively little time to encode new or lost contextual information during context (re)exposure could also have been a limiting factor on integration. Finally, participants were perhaps not motivated enough to learn during context (re)exposure, because associations between context images and angry faces are not as threatening (and therefore relevant) to the participants like context-shock pairings are to animals in a fear-conditioning paradigm. Thus, even though we confirmed that emotional memories were less dependent on context than neutral memories, the present paradigm may have obstructed in several ways the integration of new information with existing memory such that contextual dependency remained unaffected by our manipulations.

Improving the experimental paradigm with respect to these factors (more distinct memories and an incentive to learn during context (re)exposure) is particularly important in light of clinical translation. Indeed, interventions that include a return to the spatial environment in which an emotional event occurred usually introduce, like fear-conditioning research (e.g., prediction error^66^), corrective learning during treatment sessions (e.g., imaginal exposure and imagery rescripting^33,34^). Successful translation from the lab to the clinic is a notoriously big challenge. Even clinical applications that on the surface seem to have clear and straightforward experimental analogues (e.g., exposure therapy and extinction training, or cognitive restructuring and cognitive reappraisal) often contain important elements that are not accurately captured in the lab^67^. Therefore, and given the obvious differences between interventions and the paradigm we used in this study, we did not set out to model a specific form of treatment in this research. Our goal was simply to isolate the effect of a return to a context in which an emotional event occurred (and similar contexts). For the future, however, it will be important to develop ways to study how context reexposure interacts with other elements of therapeutic interventions (e.g., the offering of corrective information).

What might be a fruitful approach, as a first step, towards overcoming these major hurdles? Adopting somewhat more naturalistic methods like the trauma-film paradigm^68^ would of course increase external validity. Yet, this method does not allow experimental control over central and contextual elements of a memory, a comparison between emotional and neutral memory, or the inclusion of a large numbers of trials to accurately study the role of reliving strength during context (re)exposure, all of which are crucial for the research aims of this study. Interestingly, the Trier Social Stress Test (TSST) has recently been used to study central-object, peripheral-object, and spatial memory of an emotional event, relative to a non-stressful control episode (i.e., “friendly TSST). This allows for more experimental control within a paradigm that is relatively natural^69^. Nevertheless, this paradigm includes one trial per participant, thus also complicating the assessment of reliving effects on memory change.

Another option to consider is the episodic conditioning tool we have recently developed^70^. By presenting distressing images with a corresponding sound (e.g., an image of a broken leg together with a sound of breaking celery), a relatively rich and intense emotional experience can be induced. This is not as naturalistic as for example the adapted TSST^69^, yet still offers many advantages. First, the unique and aversive picture-sound combinations conceivably result in more distinct and well-established emotional memories than the paradigm that was adopted in this study. That is, the stimuli presented are more diverse and more distressing than the similar-looking faces. This may also help to induce learning during reexposure to stimuli (e.g., contexts) that were predictive of the aversive pictures and sounds. Indeed, the increased psychophysiological responding to predictive stimuli shows that participants anticipate threat so that one can suspect that this method elicits more incentive to learn during context (re)exposure than when angry faces are presented during learning. These psychophysiological measures could also help to connect new insights with the traditional fear-conditioning studies that the hypotheses in this study were built on and clinical interventions (i.e., analyses are not restricted to declarative knowledge data). Finally, the inclusion of many trials allows for multilevel analyses and thereby insight into subtle—yet important—factors such as reliving strength during context (re)exposure.

Apart from methodological improvements, a consideration for future research is the age of memories that are investigated. In this study, we inserted a delay of 24 h in between each experimental phase (encoding, context (re)exposure, test), to make sure that memories were consolidated (e.g., participants had slept^42^) before memory manipulation was introduced. It seems though that emotional memories, including their contextual dependency, keep changing well beyond one day after learning. For example, fear-conditioning research has shown that fear generalization tends to increase with additional passage of time^28^. Furthermore, in a recent study we have shown that contextual dependency of emotional episodic memories becomes increasingly small over the course of two weeks, relative to neutral memories^6^. Given this time-dependent transformation of emotional memory, and since therapists—almost without exception—try to interfere with emotional memories that are much older than one day, the study of remote emotional memory (including effects of context reexposure on such memories) seems of particular importance going forward.

## Conclusion

So far, the mechanisms underlying the use of context in therapy have not been formalized, nor have these been put to the test. Revisiting the site of a traumatic event has been described as an effective component of psychotherapy^32^ and animal research shows promising effects of context reexposure on fear generalization^24–31^. Nevertheless, clinical approaches typically aim to directly target the emotional hotspots of memory^71–73^, thereby potentially overlooking a complementary route towards desirable treatment outcomes. Here, we performed a first step in testing the potential of context reexposure to therapeutically alter contextual dependency of human emotional episodic memories. The present study does not provide evidence for any such effects. These null findings are not due to a lack of power or failure to replicate results that have been obtained in earlier research: Relevant Bayes factors showed moderate evidence for the null in the hypotheses tests, and assessments of contextual dependency of memory produced results that are similar to previous research. This, however, does not imply that context reexposure should no longer be viewed as a potentially viable approach to manipulate emotional memories. Methodological shortcomings (e.g., a failure to induce distinct and well-established memories) could very well have prevented the occurrence of the predicted effects. Adopting paradigms that have the potential to overcome these limitations (e.g., the episodic conditioning tool^70^) will be crucial moving forward. Doing so may improve our understanding of how contextual dependency of memory can be targeted and guide us towards successful ways to keep emotional memories at bay.

### Supplementary Information

Supplementary Figures.

## Data Availability

The datasets analyzed in this study and the analysis code have been deposited at Open Science Framework (OSF) and are available at https://osf.io/24hqa/.
