# Supplementary material for: Context reexposure to bolster contextual dependency of emotional episodic memory
Source: Sci Rep. 2023 Oct 18;13:17792. doi: 10.1038/s41598-023-40982-0 (PMC10584942; doi:10.1038/s41598-023-40982-0)
Supplement: Supplementary file 1 — Supplementary Figures. [file 41598_2023_40982_MOESM1_ESM.pdf]

Supplementary figures for:

Context reexposure to bolster contextual dependency of emotional episodic memory

Wouter R. Cox[1*], Mandy Woelk[2], Olivier T. de Vries[1], Angelos-Miltiadis Krypotos[3], Merel Kindt[1], Iris M. Engelhard[3], Dieuwke Sevenster[3], Vanessa A. van Ast[1*]

[1]Department of Clinical Psychology, University of Amsterdam, The Netherlands
[2]Research Unit Behaviour, Health, and Psychopathology, KU Leuven, Belgium
[3]Department of Clinical Psychology, Utrecht University, the Netherlands

Correspondence to: W.R.Cox@uva.nl, V.A.vanAst@uva.nl

SUPPLEMENTARY FIGURES

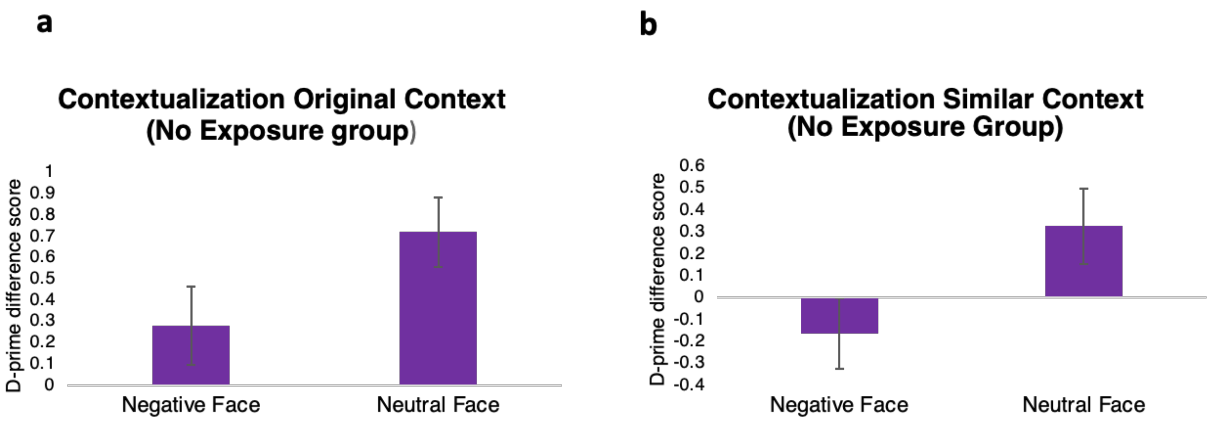

Fig. S1. (**a**) D-prime Contextualization Original Context (left) and (**b**) Contextualization Similar Context scores (right) in the No Exposure groups (separately plotted for the Negative Face and Neutral Face groups). Error bars represent SEM.

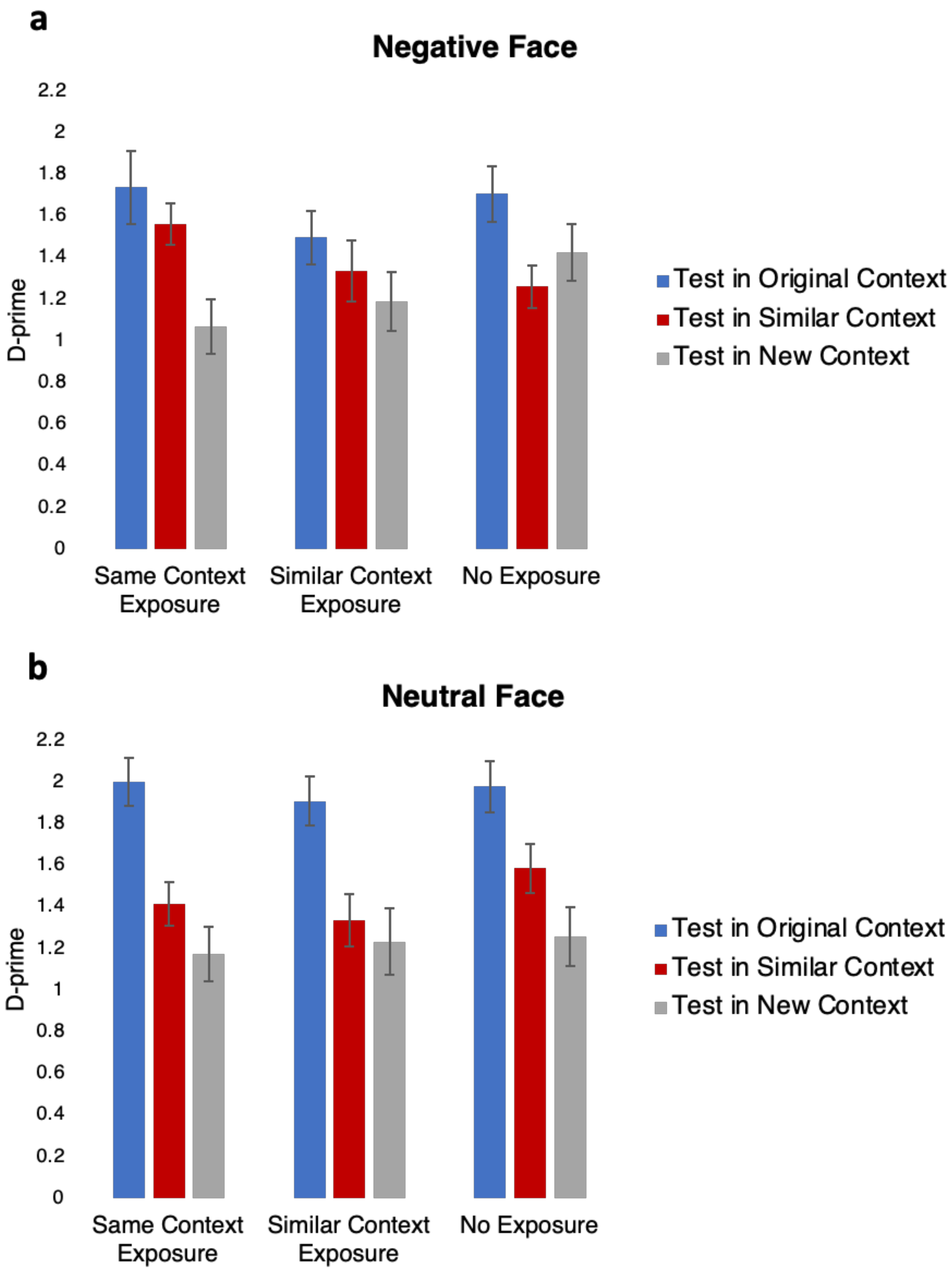

Fig. S2. D-prime accuracy scores in the Negative Face (**a**) and Neutral Face (**b**) groups for the Test in Original Context (blue), Test in Similar Context (red), and Test in New Context (grey) conditions. The scores are plotted separately for the Same Context Exposure (left), Similar Context Exposure (middle), and No Exposure (right) groups. Error bars represent SEM.

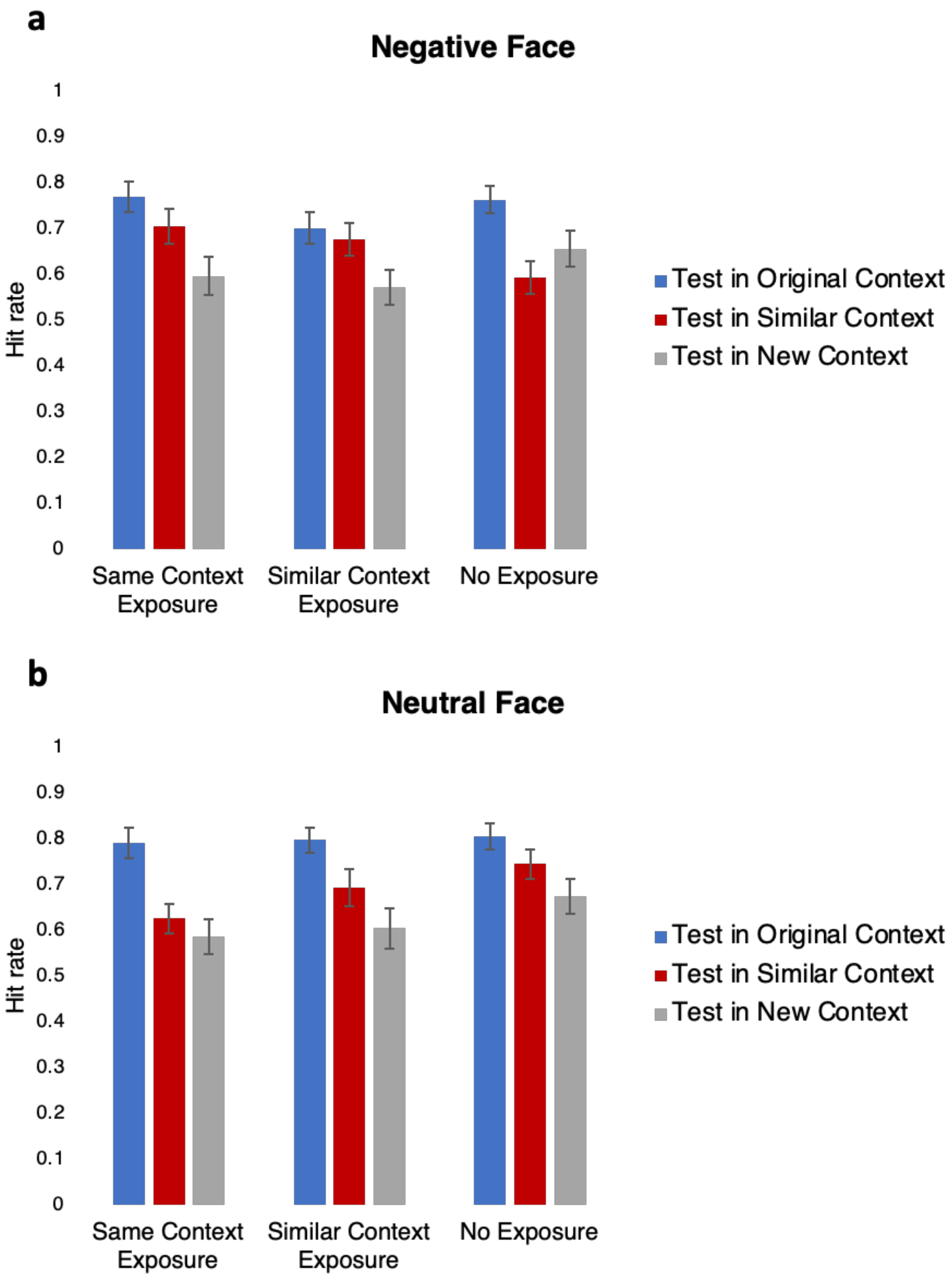

Fig. S3. Hit rates in the Negative Face (**a**) and Neutral Face (**b**) groups for the Test in Original Context (blue), Test in Similar Context (red), and Test in New Context (grey) conditions. The scores are plotted separately for the Same Context Exposure (left), Similar Context Exposure (middle), and No Exposure (right) groups. Error bars represent SEM.

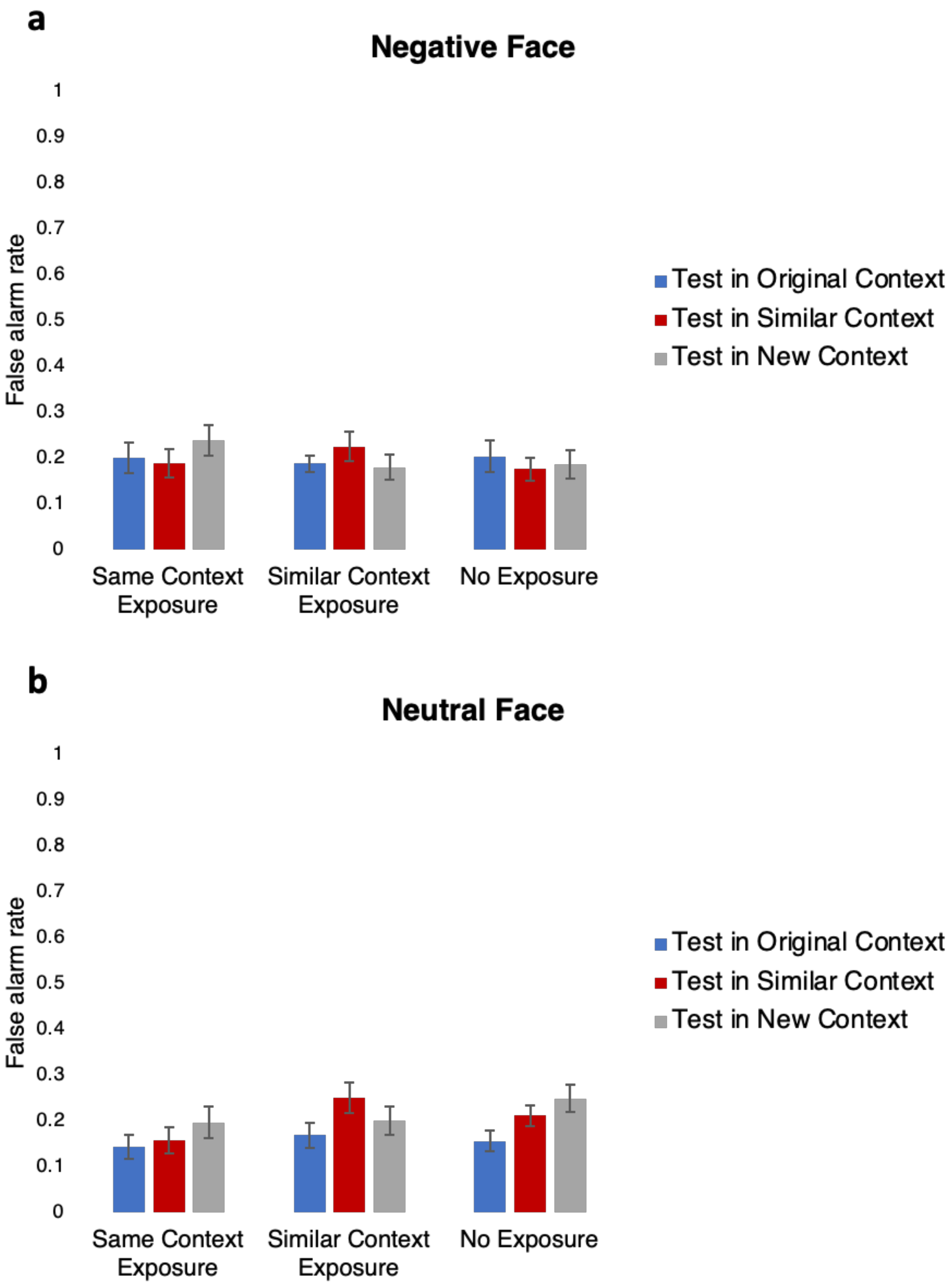

Fig. S4. False alarm rates in the Negative Face (**a**) and Neutral Face (**b**) groups for the Test in Original Context (blue), Test in Similar Context (red), and Test in New Context (grey) conditions. The scores are plotted separately for the Same Context Exposure (left), Similar Context Exposure (middle), and No Exposure (right) groups. Error bars represent SEM.
